# Neutrophil subtypes shape HIV-specific CD8 T-cell responses after vaccinia virus infection

**DOI:** 10.1038/s41541-021-00314-7

**Published:** 2021-04-12

**Authors:** Mauro Di Pilato, Miguel Palomino-Segura, Ernesto Mejías-Pérez, Carmen E. Gómez, Andrea Rubio-Ponce, Rocco D’Antuono, Diego Ulisse Pizzagalli, Patricia Pérez, Raphael Kfuri-Rubens, Alberto Benguría, Ana Dopazo, Iván Ballesteros, Carlos Oscar S. Sorzano, Andrés Hidalgo, Mariano Esteban, Santiago F. Gonzalez

**Affiliations:** 1 Institute for Research in Biomedicine, Università della Svizzera Italiana, Bellinzona, Switzerland; 2 Department of Molecular and Cellular Biology, Centro Nacional de Biotecnología-CSIC, Madrid, Spain; 3 Center for Immunology and Inflammatory Diseases, Massachusetts General Hospital, Boston, MA USA; 4 Harvard Medical School, Boston, MA USA; 5 Department of Immunology, The University of Texas MD Anderson Cancer Center, Houston, TX USA; 6 Area of Cell & Developmental Biology, Centro Nacional de Investigaciones Cardiovasculares, Madrid, Spain; 7 Max von Pettenkofer-Institute, Ludwig-Maximilians-Universität München, Munich, Germany; 8 Bioinformatics Unit, Centro Nacional de Investigaciones Cardiovasculares, Madrid, Spain; 9 Crick Advanced Light Microscopy Science and Technology Platform, The Francis Crick Institute, London, United Kingdom; 10 Institute of Computational Science, Università della Svizzera Italiana, Lugano, Switzerland; 11 Center of Integrated Protein Science Munich and Division of Clinical Pharmacology, Klinikum der Universität München, Munich, Germany; 12 Genomics Unit, Centro Nacional de Investigaciones Cardiovasculares, Madrid, Spain

**Keywords:** Adaptive immunity, Innate immunity, Vaccines, Vaccines, Virology

## Abstract

Neutrophils are innate immune cells involved in the elimination of pathogens and can also induce adaptive immune responses. Nα and Nβ neutrophils have been described with distinct in vitro capacity to generate antigen-specific CD8 T-cell responses. However, how these cell types exert their role in vivo and how manipulation of Nβ/Nα ratio influences vaccine-mediated immune responses are not known. In this study, we find that these neutrophil subtypes show distinct migratory and motility patterns and different ability to interact with CD8 T cells in the spleen following vaccinia virus (VACV) infection. Moreover, after analysis of adhesion, inflammatory, and migration markers, we observe that Nβ neutrophils overexpress the α4β1 integrin compared to Nα. Finally, by inhibiting α4β1 integrin, we increase the Nβ/Nα ratio and enhance CD8 T-cell responses to HIV VACV-delivered antigens. These findings provide significant advancements in the comprehension of neutrophil-based control of adaptive immune system and their relevance in vaccine design.

## Introduction

Neutrophils are innate immune effector cells that form the first line of defense against microbial pathogens^1^. They immediately migrate to the inflammation site and neutralize the insult in a process termed microbial sterilization^2^, which is characterized by the engulfing of pathogens, infected and apoptotic cells^3,4^, the disarming of microorganisms through neutrophil extracellular traps^5^, the generation of reactive oxygen intermediates^6^, and the release of lytic enzymes from their granules^2^. Neutrophils can also induce antigen-specific T-cell responses by migrating to the lymph nodes (LN) and bone marrow^7,8^ where they efficiently cross-prime naive T cells^9^.

Neutrophils polarize to distinct phenotypes in response to environmental signals^10^, and in the presence of granulocyte macrophage-colony stimulating factor (GM-CSF) and interferon (IFN)-γ, neutrophils can acquire antigen-presenting cell (APC) functions and trigger CD8 T-cell activation^11^. Recent studies characterized distinct functional neutrophil subsets in cancer and infection contexts^12–14^. The cytokine/chemokine environment in tumor tissue polarizes tumor-associated neutrophils (TAN) to control tumor angiogenesis and growth^15^. In the presence of transforming growth factor (TGF)-β, TAN polarize from the anti-tumoral N1 to the pro-tumoral N2 phenotype, characterized by impaired ability to activate CD8 T cells^16^.

During virus infection, pro-inflammatory milieu can induce specific neutrophil subtypes^13^. By manipulating the vaccinia virus (VACV) vector, robust NFκB activation generates and alters neutrophil recruitment to the infection site in mice^14,17^. Distinct neutrophil subtypes (classified as Nα and Nβ) are recruited as a result of the cytokine/chemokine profile produced. Nβ cells overexpress costimulatory molecules CD80 and CD86 and show a greater capacity to generate VACV-specific CD8 T-cell responses than Nα cells in vitro^14^.

Enhanced neutrophil trafficking from the infection site to secondary lymphoid organs correlates with increased CD8 T-cell responses to HIV vector-delivered antigens^14^. Conversely, study of vaccine immunization showed that neutrophils generate poor antigen-specific CD4 responses, as their contacts with dendritic cells (DC) in the draining LN are very brief, and DC–T-cell interactions are improved in neutropenic mice^18^.

The balance between Nα and Nβ neutrophil subtypes could influence the generation of antigen-specific CD8 and CD4 T-cell immune responses and then improve the virus-based vaccine efficacy. However, the ability of neutrophil subtypes to relocate to secondary lymphoid organs, their migratory patterns, and their capacity to regulate antigen-specific CD8 T-cell responses have not been evaluated in vivo.

Attenuated VACV vectors are considered promising HIV vaccine candidates and several strategies have been developed to improve immunogenicity to HIV antigens^19^. Even if VAVC vector approaches gave encouraging results in primates^20^, the effectiveness of RV144 phase III HIV/AIDS human clinical trial was limited^21^. Given the importance of understanding the mechanism of neutrophil-dependent control of T-cell subset reactions to virus-delivered antigens and the interest for the generation of vaccines that preferentially induce HIV-specific CD8 T-cell responses, in this work we studied those processes in secondary lymphoid organs.

Here, we characterized Nα and Nβ splenic migratory features by multiphoton intravital microscopy (MP-IVM) and we detailed the transcriptomic profiles of these distinct neutrophil populations by RNA-sequencing and flow cytometry. Finally, we found an alternative approach to increase the antigen-specific CD8 T-cell responses. Indeed, since Nβ neutrophils compared to Nα overexpressed α4β1 integrin, also called very-late-antigen (VLA-4), administration of low dose of anti-VLA-4 increased the Nβ/Nα ratio in the peritoneal cavity (PEC) and spleen and in turn augmented the HIV-specific CD8 T-cell responses. These findings provide important insights into the neutrophil-dependent mechanism of the poxvirus-induced immune response and offer alternative strategies for HIV vaccine vector design.

## Results

### HIV-specific T-cell responses are generated in the spleen after neutrophil accumulation

**Fig. 1:**
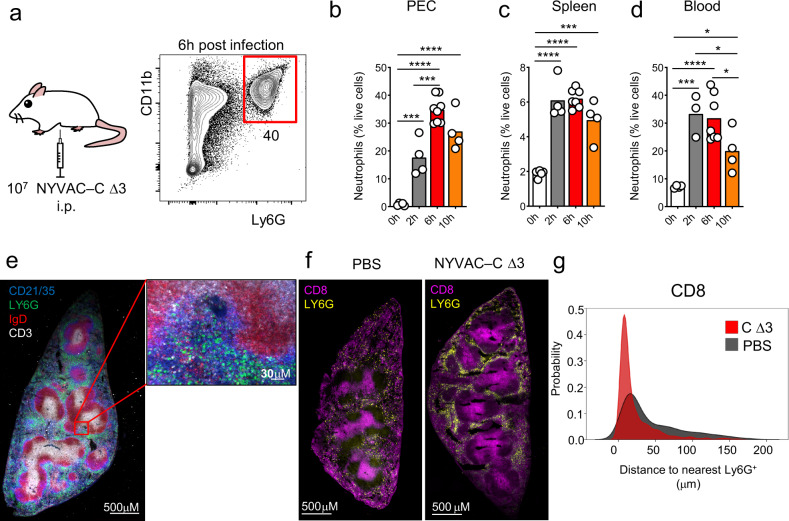
In vivo time-course of neutrophil accumulation after poxvirus infection. BALB/c mice were infected by intraperitoneal (i.p.) injection of 10^7^ PFU of NYVAC-C Δ3 virus and Ly6G^+^ CD11b^+^ neutrophils were measured at 6 h post infection (**a**). Percentages of neutrophils in peritoneal cavity (PEC) (**b**), spleen (**c**), and blood (**d**) at 0, 2, 6, and 10 h post infection from NYVAC-C Δ3 injected mice. Spleen tissue section and its magnification from NYVAC-C Δ3-infected mice. Ly6G^+^ neutrophils in green, CD3^+^ T cells in white, IgD^+^ B cells in red, and CD21/35^+^ cells in blue (**e**). Spleen tissue section from PBS and NYVAC-C Δ3-injected mice. Ly6G^+^ neutrophils in yellow, CD8^+^ T cells in purple (**f**). Distance of the nearest Ly6G^+^ cell from CD8^+^ cells. The nearest neighbor euclidean distance to Ly6G^+^ is expressed as probability density for each cell belonging to CD8^+^ clusters (**g**). Graphs show mean; each point represents an individual mouse. Data are representative of two independent experiments. *P < 0.05, ***P < 0.001, ****P < 0.0001.

To define the dynamic recruitment of neutrophil from the blood to the PEC and to the spleen, BALB/c mice were infected by intraperitoneal (i.p.) injection of 10^7^ plaque-forming units (PFU) of VACV strain NYVAC-C ΔA52RΔB15RΔK7R deletion mutant (also termed triple deletion mutant, NYVAC-C Δ3 or C Δ3) and we identified by flow cytometry Ly6G^+^CD11b^+^ neutrophils (Fig. 1a). To determine how neutrophil trafficking occurs from the primary site of infection to the spleen, we quantified neutrophils in PEC, spleen, and blood at 2, 6, and 10 h post infection (p.i.) (Fig. 1b–d). Neutrophils started to accumulate in PEC, spleen and blood after 2 h p.i. compared to PBS-treated mice and they decreased at 10 h p.i. in all cases (Fig. 1b–d). After infection, in the spleen, Ly6G^+^ neutrophils were located close to the CD21/35^+^ areas in the marginal zone and in the proximity of the CD3^+^ T-cell zone (Fig. 1e). NYVAC-C Δ3-infected mice specifically showed neutrophil accumulation surrounding the CD8^+^ areas (Fig. 1f). Indeed, the probability of CD8^+^ cells to be located close to neutrophils was significantly higher in the infected mice compared to those injected with PBS (Fig. 1g).

**Fig. 2:**
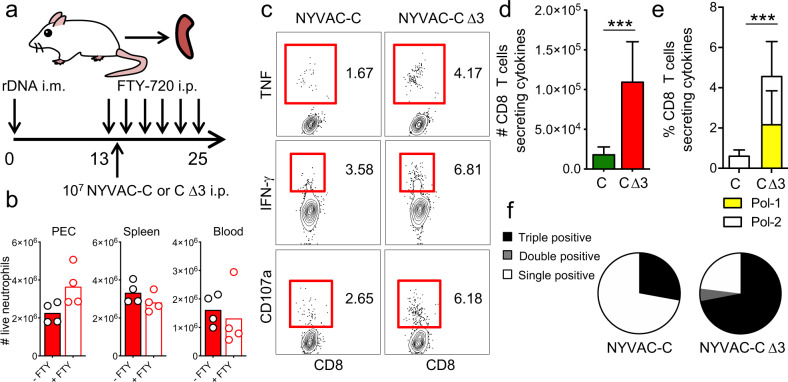
Splenic HIV-specific T-cell responses is increased after NYVAC-C Δ3 infection. Vaccine-induced HIV-1-specific CD8 T-cell responses from spleen of mice (*n* = 4 per group) primed intramuscularly with recombinant DNA, infected with 10^7^ PFUs of NYVAC-C or NYVAC-C Δ3 and injected with FTY720 every other day starting from the day before infection **(a)**. Neutrophil total numbers in peritoneal cavity (PEC), spleen, and blood of NYVAC-C Δ3-injected and FTY720- or PBS-injected mice (**b**). The response was measured 11 days after the last immunization, after splenocyte stimulation with HIV-1 peptides. Contour plots of CD8 T cells that produce IFN-γ, TNF, and CD107a after NYVAC-C or NYVAC-C Δ3 infection **(c)**. Total numbers **(d)** or percentages of Pol-1-, Pol-2-specific **(e)** CD8 T cells that express IFN-γ and/or TNF and/or CD107a. Nonspecific responses of mice infected with control NYVAC-WT were subtracted from the total value. Graphs show mean ± confidence interval (CI). Pie chart colors indicate the relative percentage of cells producing three (black), two (gray), or one (white) activation markers **(f)**. Data are representative of two independent experiments. ***P < 0.001.

We previously demonstrated that neutrophils are involved in the enhanced HIV-specific T-cell responses of NYVAC-C Δ3-infected mice compared to those infected with parental NYVAC-C^14^. To study the ability of NYVAC-C Δ3 to induce an antigen T-cell response specifically in the spleen, we used a DNA intramuscular prime/poxvirus i.p. boost approach and we concurrently blocked lymphocyte egress from secondary lymphoid organs using the functional S1P receptor antagonist FTY720 (Fig. 2a) without interfering with neutrophil accumulation in the organ of interest (Fig. 2b). This heterologous immunization protocol is more immunogenic than a homologous combination in generating T-cell responses against HIV antigens^22^. To study the HIV-specific T-cell response, splenocytes from infected mice were stimulated with HIV-1 Pol-1, or Pol-2 peptides, which are the most immunogenic MHC class I-restricted cytotoxic T lymphocytes (CTLs) peptides of HIV clade C in BALB/c mice^23^. To determine the functional profile of CD8 T cells, we measured the levels of IFN-γ, TNF, and lysosomal-associated membrane protein-1 (LAMP-1 or CD107a) as a surrogate marker for induction of killing (Fig. 2c).

We quantified the magnitude of the T-cell response as the number and percentage of T cells that expressed IFN-γ and/or TNF and/or CD107a and the polyfunctionality of the response as the capacity to express more than one of these activation markers. We showed that, compared to the parental strain, the triple deletion mutant induced a robust increase of the quality of HIV-specific CD8 T-cell responses since NYVAC-C Δ3 injected-mice had higher number and percentage and even more polyfunctional HIV-specific CD8 T cells (Fig. 2d–f).

These findings show that antigen-specific T-cell response was induced and increased in the spleen after NYVAC-C Δ3 infection and correlated with a robust neutrophil infiltration in this lymphoid organ.

### Neutrophil subtypes display distinct dynamic behaviors in the spleen

After NYVAC-C Δ3 i.p. infection, PEC of C57BL/6 J mice showed two neutrophil populations (Nα and Nβ) that differed in Ly6G/CD11b expression, size, and complexity, and expressed Ly6B.2 similarly at high levels (Supplementary Fig. 1a). Compared to neutrophil precursors (preNeu), immature and mature neutrophils from bone marrow (Supplementary Fig. 1b), peritoneal Nα and Nβ show levels of CXCR4 and CXCR2 similar to mature neutrophils (Supplementary Fig. 1c). Bulk RNA sequencing analysis reveals that Nα neutrophils are quite dissimilar from Nβ (Supplementary Fig. 1d). Indeed, these two subsets showed vast transcriptomic differences and had 2655 differentially expressed genes (Supplementary Fig. 1e). Furthermore, analysis of genes differentially expressed by tumor-associated neutrophils and mostly overexpressed by N1 compared to N2^24^ shows that Nβ downregulated many of those genes in comparison to Nα (Supplementary Fig. 1f).

**Fig. 3:**
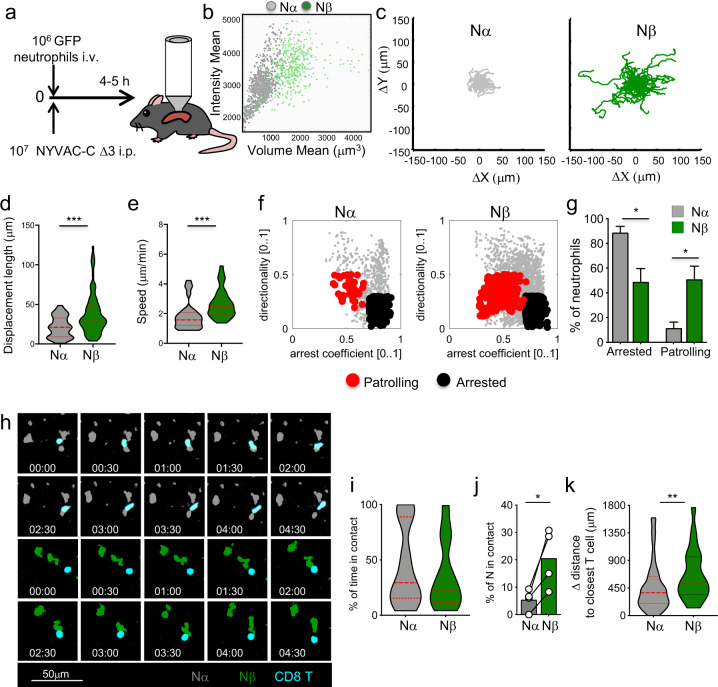
Distinct dynamic behaviors from different neutrophil subtypes. 10^6^ GFP^+^ neutrophils isolated from UBC-GFP mice were intravenously (i.v.) injected in C57BL6/J mice that were i.p. infected with 10^7^ PFU of NYVAC-C Δ3 virus and the spleen was imaged from 4 to 5 h post infection (**a**). Multiphoton intravital microscopy dot plot determined by intensity and volume mean of GFP^+^ neutrophils from spleen of NYVAC-C Δ3-infected mice (**b**). Cell track plot of Nα and Nβ neutrophil from spleen of NYVAC-C Δ3 infected mice from 4 to 5 h post infection (**c**). Violin plots of displacement length (μm) (**d**) and speed (μm/min) (**e**) of Nα and Nβ neutrophils from 4 to 5 h post infection. Dot plots based on directionality and arrest coefficient of neutrophil subset activities (arrested: black; patrolling: red) (**f**). Percentages of arrested and patrolling Nα and Nβ from 4 to 5 h post infection (**g**). Nα (gray), Nβ (green) neutrophil and cognate CD8 T cells (cyan) from spleen of NYVAC-C Δ3-infected mice from 4 to 5 h post infection (**h**). Violin plots of percentage of time in contact of Nα and Nβ (**i**), graph bars of percentage of Nα and Nβ in contact with CD8 T cells (**j**), and violin plots of differential distance of Nα and Nβ to closest T cell (**k**). Graphs show mean ± SEM; each point represents an individual mouse. Data are representative of three independent experiments. *P < 0.05, **P < 0.01, ***P < 0.001.

Compared to 2 h p.i., the ratio Nβ/Nα significantly increased in the PEC, spleen, and blood at 6 h and quickly decreased at 10 h after infection (Supplementary Fig. 1g–i), suggesting that the time window between 2 and 10 h represents an opportunity for Nβ to be involved in processes of immune regulation. We tested whether Nα and Nβ showed distinct dynamic behaviors in vivo in the spleen within this range of time. GFP^+^ neutrophils isolated from UBC (ubiquitin C)-GFP mice were intravenously (i.v.) injected in NYVAC-C Δ3-infected mice and analyzed by splenic MP-IVM from 4 to 5 h after injection (Fig. 3a). By the tracked mean volume of GFP^+^ cells, we could distinguish two neutrophil populations that differed in volume (Fig. 3b) and showed comparable sizes to the Nα and Nβ populations previously described^14^. The bigger population in green (Nβ) was not found in the PBS-injected mice (data not shown) as we previously showed^14^.

We analyzed these neutrophil subtypes and found that Nβ cell tracks (in green) showed an increased migratory pattern compared to Nα cell tracks (in gray) (Fig. 3c and Supplementary Videos 1 and 2). Moreover, the displacement length as well as the speed of Nβ were significantly higher than Nα between 4 and 5 h p.i. (Fig. 3d, e). To understand if these different neutrophil subsets have distinct biological functions, we analyzed their migratory patterns based on their directionality and arrest coefficient^25^. Indeed, we found that Nβ neutrophils had distinct migratory pattern compared to Nα subset between 4 and 5 h p.i. (Fig. 3f). Nβ were significantly more patrolling cells than Nα, which conversely showed a significantly more arrested behavior between 4 and 5 h p.i. (Fig. 3g). Accordingly, compared to Nα, Nβ upregulated several G protein-coupled receptors (GPCR) genes associated with cell migration, and specifically those belonging to the C and adhesion classes (Supplementary Fig. 2).

To test whether these distinct motility and migratory patterns correlate with dissimilar neutrophil ability to interact with cognate CD8 T cells, labeled CD8 T cells from poxvirus-infected mice were adoptively transferred together with GFP neutrophils in recipient mice that were NYVAC-C Δ3-infected. Neutrophil and CD8 T-cell interactions were analyzed and quantified by splenic MP-IVM between 4 and 5 h p.i. (Fig. 3h and Supplementary Videos 3 and 4). Both subtypes showed interactions with a similar duration (less than 5 mins) and spent comparable percentage of imaged time in contact with CD8 T cells (Fig. 3h, i). Nevertheless, Nβ showed an increased ability to interact and find CD8 T cells compared to Nα as evidenced by a higher percentage of Nβ that established CD8 T-cell contacts (Fig. 3j) as well as their increased capacity to travel longer distances to get closer to CD8 T cells (Fig. 3k and Supplementary Videos 3 and 4).

These results show that Nβ neutrophils were more motile and prone to establish CD8 T-cell contacts than Nα in secondary lymphoid organs after NYVAC-C Δ3 infection.

### Neutrophil subtypes show a different expression of α4β1 integrin

To understand better the different dynamics of recruitment of both neutrophil subsets from the primary site of infection to secondary lymphoid organs, we analyzed the expression of the surface markers involved in neutrophil migration and adhesion, such as IL-1 receptor (IL-1R), CXCR2, and very-late-antigen (VLA-4) α4β1. IL-1R has a clear role in neutrophil recruitment to the peritoneal cavity^26^ and migration to secondary lymphoid organs^25^. CXCR2 regulates neutrophil release from bone marrow^27^ and neutrophil migration in tumor microenvironment and during severe sepsis^28,29^; it has been shown that failure of CXCR2 downregulation controls neutrophil mobilization during inflammation in the peritoneum^30^. VLA-4 α4β1 integrin is involved in leukocyte transendothelial migration^31^ and neutrophils use this integrin in the rolling phase during the inflammatory response^32^.

**Fig. 4:**
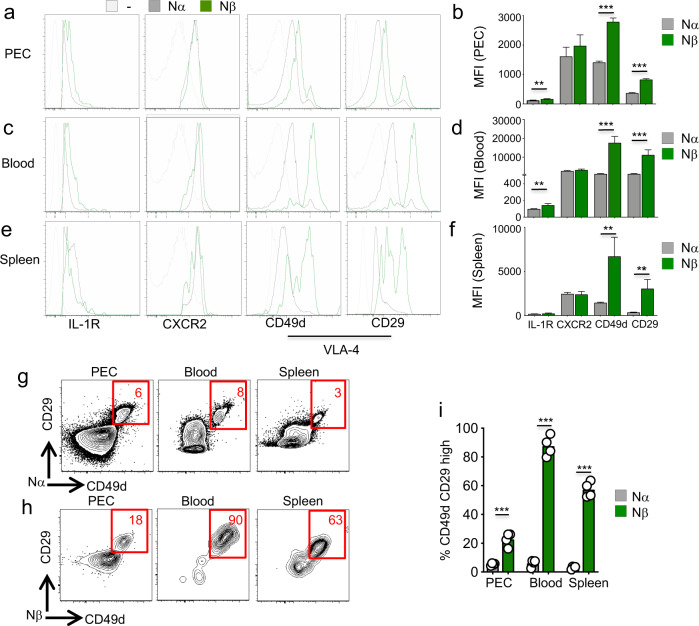
Different expression of VLA-4 integrin by distinct neutrophil subsets. Layouts and median fluorescence intensity (MFI) of IL-1R, CXCR2, CD49d, and CD29 in Nα (gray) and Nβ (green) neutrophils at 6 h post infection in PEC (**a**–**b**), blood (**c**–**d**), and spleen (**e**–**f**) of NYVAC-C Δ3-injected mice. Isotype control in gray. Columns show mean ± SEM of four mice. Contour plots of CD29 and CD49d markers of Nα (**g**) and Nβ (**h**) neutrophils from PEC, blood, and spleen of NYVAC-C Δ3-injected mice at 6 h post infection. Percentages of CD49d and CD29 high Nα (gray) and Nβ (green) neutrophils from PEC, blood, and spleen of NYVAC-C Δ3-injected mice (**i**). Graphs show mean; each point represents an individual mouse. Data are representative of three independent experiments. **P < 0.01, ***P < 0.001.

Nβ in the peritoneal cavity of NYVAC-C Δ3-infected C57BL/6 J mice had similar levels of CXCR2 compared to Nα (Fig. 4a) but they started to overexpress IL-1R and CD49d (α4) CD29 (β1) integrins. Indeed, the levels of these last surface markers were significantly higher in Nβ than in Nα neutrophils (Fig. 4b). In the blood, we detected no difference in CXCR2 levels between these populations; however, Nβ upregulated CD49d and CD29 compared to surface levels of these cells in the PEC (Fig. 4c). By analyzing the surface expression of these markers, we found that Nβ expressed more IL-1R and VLA-4 than Nα did (Fig. 4d). Finally, in the spleen, Nβ showed the same levels of IL-1R and CXCR2 as Nα (Fig. 4e); however, they still significantly overexpressed VLA-4 compared to Nα, even if VLA-4 expression levels were lower than those in the blood (Fig. 4e, f).

A deep analysis of VLA-4 expression in those organs showed that Nα almost uniformly expressed this integrin at low levels, while a fraction of Nβ was highly positive for this marker (Fig. 4g, h). Indeed, Nβ were around 20% highly positive cells in the PEC, they were almost homogenously highly VLA-4 expressing cells in the blood, and still 60% highly positive cells in the spleen (Fig. 4h). Overall, the percentages of highly positive VLA-4 Nβ cells were significantly higher than Nα in all the tested organs (Fig. 4i).

These data indicate that IL-1R was upregulated in PEC and blood, while VLA-4, which is involved in transendothelial migration, was predominantly overexpressed in blood and spleen. In addition, VLA-4 was ubiquitously at higher levels in Nβ neutrophils compared to Nα to possibly promote their trafficking from and towards secondary lymphoid organs.

### α4β1 integrin inhibitor enhances CD8 T-cell responses by increasing Nβ/Nα ratio

**Fig. 5:**
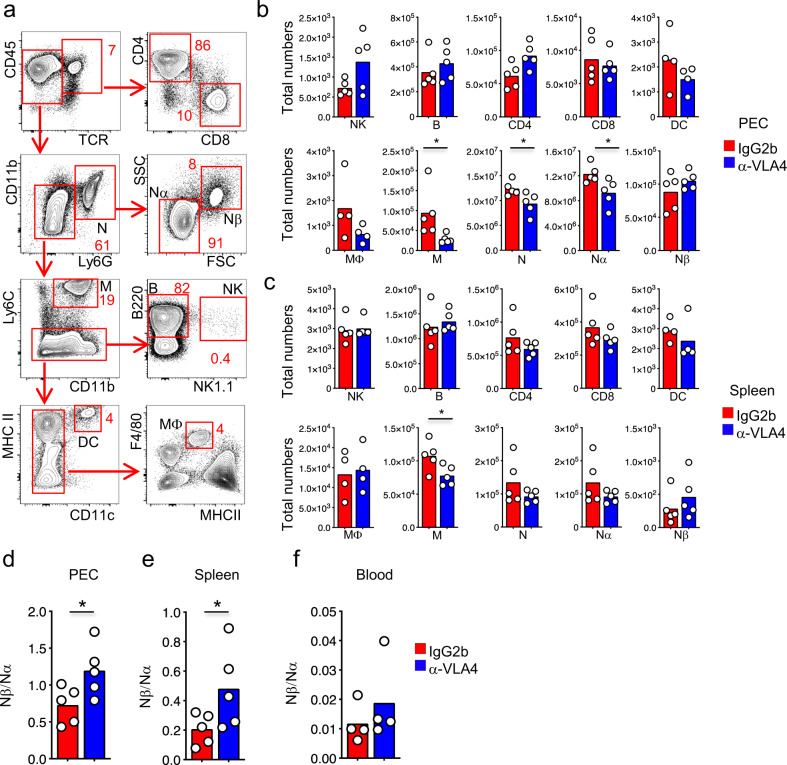
Inhibition of VLA-4 increases Nβ/Nα ratio. Contour plot composition of peritoneal immune infiltrate after NYVAC-C Δ3 infection. Proportion of TCR^+^CD4^+^ and TCR^+^CD8^+^ T cells, CD11b^+^ Ly6G^+^ neutrophils divided by SSC and FSC in Nα and Nβ, CD11b^+^ Ly6G^−^ Ly6C^+^ monocytes (M), B220^+^ NK1.1^−^ B cells, NK1.1^+^ NK cells, MHCII^+^ CD11c^+^ dendritic cells (DC) and F4/80^+^ MHCII^+^ macrophages (MΦ) were measured at 6 h post infection (**a**). Total numbers of NK, B, CD4, CD8, DC, MΦ, M, N, Nα, and Nβ from PEC (**b**) and spleen (**c**) of NYVAC-C Δ3 infected and IgG2b or anti-VLA4 antibody-treated mice. Mice were injected with IgG2b isotype or anti-VLA4 antibody 16 h before- and 1 h post infection. Nβ/Nα ratio from PEC (**d**), spleen (**e**), and blood (**f**) of infected and IgG2b or anti-VLA4 antibody-treated mice. Graphs show mean; each point represents an individual mouse. Data are representative of two independent experiments. *P < 0.05.

To determine whether the different expression of α4β1 VLA-4 integrin by distinct neutrophil subtypes results in the possibility to manipulate their infiltration in the primary site of infection and the secondary lymphoid organ, we injected mice with a low dose (250 μg) of α4β1 antagonist Natalizumab (monoclonal anti-VLA-4) that controls the migration of immune cells^33^. We broadly characterized the major immune subsets that could express α4β1 integrin (Fig. 5a). We quantified the abundance of these immune subsets in the PEC and in the spleen of mice that were treated with IgG2b isotype antibody or anti-VLA4 antibody and infected with 10^7^ PFU of NYVAC-C Δ3. With the exception of monocytes (M) and neutrophils (specifically Nα), the total numbers of CD4, CD8, NK, B, DC, and macrophages (Mϕ) were similar in the PEC and spleen of IgG2b- or anti-VLA4 antibody-treated mice at 6 h p.i. (Fig. 5b, c). Conversely, there were no differences in Nβ neutrophil total numbers (Fig. 5b, c). As a result of this distinct neutrophil accumulation, the ratio Nβ/Nα was significantly increased in the PEC and spleen of those mice treated with α4β1 inhibitor, and a similar trend was observed in the blood (Fig. 5d–f).

**Fig. 6:**
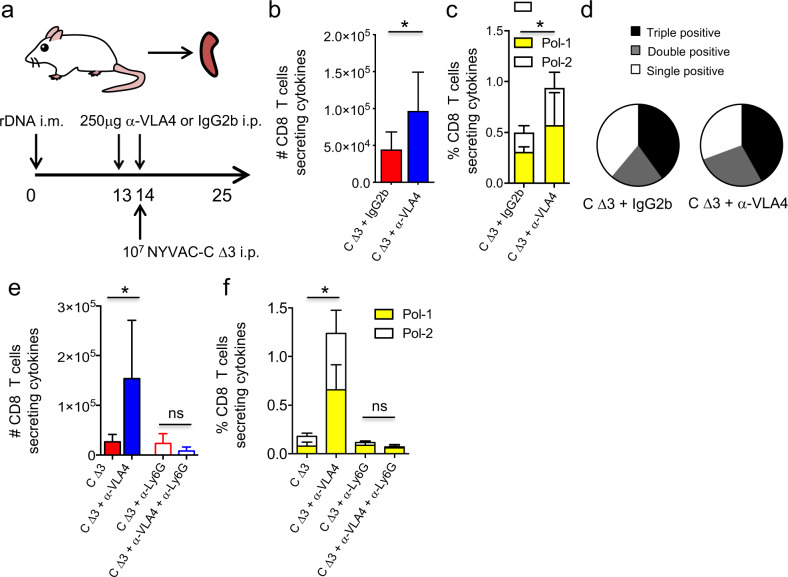
Inhibition of VLA-4 increases HIV-specific T-cell responses. Vaccine-induced HIV-1–specific CD8 T-cell response from spleen of mice (*n* = 4 per group) primed intramuscularly with recombinant DNA, infected with 10^7^ PFUs of NYVAC-C Δ3 and injected with IgG2b isotype or anti-VLA4 antibody 16 h before and 1 h post infection (**a**). The response was measured 11 days after the last immunization, after splenocyte stimulation with HIV-1 Pol-1/Pol-2 peptides. Total numbers (**b**) or percentages of Pol-1-, Pol-2-specific (**c**) CD8 T cells that express IFN-γ and/or TNF and/or CD107a. Nonspecific responses of mice infected with control NYVAC-WT were subtracted from the total value. Graphs show mean ± SD. Pie chart colors indicate the relative percentage of cells producing three (black), two (gray), or one (white) activation markers (**d**). Total numbers (**e**) or percentages of Pol-1-, Pol-2-specific (**f**) CD8 T cells that express IFN-γ and/or TNF and/or CD107a in mice infected with 10^7^ PFUs of NYVAC-C Δ3, injected with IgG2b isotype or anti-VLA4 and treated or untreated with anti-Ly6G. *P < 0.05.

To test whether the increase of Nβ/Nα ratio in anti-VLA4 treated mice could influence the CD8 adaptive immune responses, BALB/c mice were immunized following the previously described heterologous immunization protocol (DNA prime/ NYVAC-C Δ3 boost) and treated with IgG2b isotype or anti-VLA4 antibody. CD8 T-cell responses to HIV Pol-1/Pol-2 antigens were analyzed from spleen (Fig. 6a). We quantified the magnitude of the T-cell responses as the number and percentage of T cells secreting IFN-γ and/or TNF and/or CD107a and the polyfunctionality as the capacity to express more than one of these markers. We observed in NYVAC-C Δ3-infected mice that, compared to IgG2b-treated mice, the inhibition of α4β1 integrin and infection with NYVAC-C Δ3 induced a significant increase of the magnitude of the HIV-specific CD8 T-cell responses (Fig. 6b, c), while the polyfunctionality was similar (Fig. 6d).

To demonstrate whether the increase of the magnitude of T-cell responses in mice treated with anti-VLA4 antibody was neutrophil-dependent, we depleted these cells in vivo using anti-Ly6G mAb (1A8), administered 24 h before virus boost. Compared to control, mice treated with anti-VLA4 showed significant increase in total numbers and percentage of the Pol-1–, Pol-2–specific CD8 T-cell responses only when neutrophils were present (Fig. 6e, f). Indeed, mice injected with anti-Ly6G show low and similar CD8 T-cell response if were treated with or without anti-VLA4 (Fig. 6e, f).

**Fig. 7:**
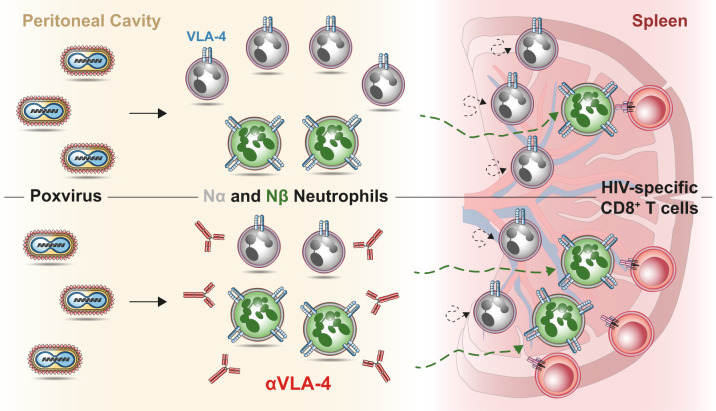
Model of action. After poxvirus infection in the peritoneal cavity, two distinct subtypes of neutrophil (Nα and Nβ) are generated. Nβ are characterized by overexpression of the trans-endothelial migration marker VLA-4 and an increased ability to interact with antigen-specific CD8^+^ T cells in the spleen (top panel). Poxvirus infection in combination with VLA-4 inhibition increases Nβ/Nα ratio in peritoneal cavity and spleen leading to augmented HIV-specific CD8 T-cell responses (bottom panel).

These findings show that HIV-specific CD8 T-cell response was enhanced whenever we increased Nβ/Nα ratio. Hence, systemic inhibition of α4β1 integrin, by shaping neutrophil subsets accumulation in primary site of infection and secondary lymphoid organs and by increasing antigen-specific CD8 T-cell responses (Fig. 7), provides new opportunities for vaccine design.

## Discussion

In this study we described the in vivo accumulation of distinct neutrophil subsets in the primary site of VACV infection, their capacity to relocate to secondary lymphoid organs where CD8 T cells adaptive and memory responses take place. Furthermore, we characterized several markers associated with their ability to migrate to different tissues and defined, by shaping their ratio, a new strategy to increase HIV-specific CD8 responses that could be successfully applied in the design of new poxvirus-based vaccines.

Attenuated poxvirus vectors are one of the best vaccine candidates and, specifically, MVA and NYVAC poxvirus strains are used against cancer and infectious diseases in humans^34^. With respect to VACV vectors towards Acquired Immune Deficiency Syndrome (AIDS), several approaches have been developed to improve their immunogenicity to HIV antigens^19^. Among those, innate immune stimulation showed a clear therapeutic effect in monkeys infected with simian immunodeficiency virus^35^. So far, among all the clinical trials carried out with distinct AIDS vaccine candidates, only the RV144 phase III showed modest protection^21^. Nonetheless, new MVA/NYVAC-based clinical trials have been recently performed with promising results^36–38^. Based on these studies, there is major interest in understanding the VACV vectors-based mechanisms of induction of T-cell responses and, in turn, in increasing the effectiveness of those vaccines.

In this regard, NYVAC-C ΔA52RΔB15RΔK7R deletion mutant showed to be able to enhance HIV antigen-specific CD8 T cellular and humoral immune responses compared to many other NYVAC-C viruses. The mechanism of action is related with the targeting of the NFκB central host-cell signaling pathway and with the increasing of neutrophil migration in the peritoneal cavity and spleen of infected mice^14,17^. Upon infection, neutrophil characterization revealed two distinct cell size subsets that showed different APC properties and capacities to generate VACV-specific CD8 T-cell responses in vitro^14^.

Here, in order to extend those previous findings, we better characterized these neutrophil subsets by defining their transcriptional profile. Bulk RNA-sequencing analysis showed clearly two distinct neutrophil subpopulations: Nα upregulated more than 2000 genes compared to Nβ, and among those several N1 genes. A previous study^39^ defined N1 and N2 markers but a more recent RNA-sequencing analysis^24^ highlighted 138 genes with the strongest alteration between N1 and N2. Among those, 136 genes were downregulated by N2 neutrophils and approximately 100 of those genes were downregulated by Nβ cells in our study. Together, these data indicate a transcriptomic similarity between N2 and Nβ and between N1 and Nα neutrophils. Further studies, where neutrophil subsets isolated from two different environments (tumor and infection) are contemporaneously RNA-sequenced, have to demonstrate how similar these neutrophil subpopulations are.

Additionally, we defined how both neutrophil subtypes behave in the spleen and showed that Nβ moved faster, migrated more, and spend more time patrolling than Nα. Nβ higher speed and patrolling ability could facilitate the interactions of this neutrophil subset with HIV-specific T-cell clones. Indeed, a recent study also distinguished two subsets of splenic Ly6G^+^ cells, immature and mature neutrophils, that moved differently in the spleen and contributed differentially to the eradication of *Streptococcus pneumoniae*^40^. In this direction, our in vivo movies show how Nβ move towards CD8^+^ T cells and they establish short interactions more frequently than Nα neutrophils. Further studies will have to specifically address how these Nβ–T-cell interactions and alternative mechanisms, such as release of factors from Nβ in the T-cell proximity, might be regulating T-cell activation/proliferation.

The higher expression levels of VLA-4 by Nβ cells and their larger percentages of highly positive cells compared to Nα, indicate that Nβ have advantage in cell recruitment and displacement. Indeed, the role of VLA-4 has been previously shown in neutrophil recruitment^32^, and by blocking VLA-4 it is possible to reduce neutrophil infiltration and limit the neuronal injury from ischemic stroke^41^. Along the same lines, here, a low dose of VLA-4 inhibitor decreased the amount of VLA-4^low^ Nα cells that infiltrated the peritoneal cavity and the spleen, without limiting the recruitment of VLA-4^high^ Nβ neutrophils. As a consequence, in the presence of the VLA-4 inhibitor, Nβ/Nα ratio was increased in both tissues. High doses of VLA-4 inhibitor have been shown to reduce the amounts of lymphocytes^42^. However, in our study, the mice that received a low dose of antibody had comparable CD4 and CD8 T cell total numbers to those mice that did not receive the inhibitor.

The increase of Nβ/Nα ratio correlated with a higher HIV-1 Pol-1/Pol-2-specific CD8 T-cell responses in mice that received the VLA-4 α4β1 integrin inhibitor. Pol is one of the best conserved HIV-1 proteins^43^ and increased number of CD8 response from variable Env epitopes to Pol epitopes is associated with control of viremia in the first years of HIV-1 infection^44^. Pol-specific CD8 T-cell response in NYVAC-C Δ3-infected and anti-VLA-4 treated mice is increased and is still mainly polyfunctional compared to those mice only infected with NYVAC-C Δ3. Considering that human HIV non progressors preferentially maintain highly functional HIV-specific CD8 T cells^45^, the NYVAC-C Δ3 vaccinia vector in combination with α4β1 integrin inhibitor might result in a promising approach for HIV prophylaxis and AIDS treatment.

In summary, here we describe how neutrophils, after poxvirus infection, accumulate in the primary site of infection and close to CD8 T cells on secondary lymphoid organs. Two distinct neutrophils subsets display different migratory patterns that correlate with distinct levels of markers associated with their recruitment. Moreover, by inhibiting α4β1 integrin and manipulating the migration of these neutrophil subsets in the peritoneal cavity and in the spleen, we increased the vaccine efficacy of NYVAC-C vector in terms of CD8 T-cell adaptive immune responses specific for HIV heterologous antigens. Our findings could be relevant to develop a valid prototype for future HIV vaccine and provide novel basis for virus vaccine vector design.

## Methods

### Mice and injections

BALB/c and C57/BL6J mice (6–8 weeks old) were purchased from Harlan and Janvier labs. UBC (ubiquitin C)-GFP mice with a C56BL/6 background^46^ were bred in-house. NYVAC-C vector was provided by Sanofi Pasteur and NYVAC-C Δ3 was previously described^14^. For the DNA prime immunization protocol to assay virus immunogenicity, mice received 100 μg of DNA-C (50 μg pcDNA-_CN54_GP120 + 50 μg pcDNA-_CN54_GagPolNef) or 100 μg DNA-ϕ (pcDNA) by the intramuscular route^22^. Plasmids were purified using Endofree Plasmid Mega Kit (Qiagen). After two weeks, mice were immunized i.p. with 10^7^ PFU of NYVAC-WT, NYVAC-C, or NYVAC-C Δ3. Mice immunized with sham DNA-ϕ prime and NYVAC-WT boost were used as controls. FTY720 (1 mg kg^−1^ bodyweight) in 150 μl H_2_O was injected intraperitoneally every other day until the end of the experiment. Anti-VLA-4 antibody (PS/2, BioXcell) or IgG2b, κ isotype control antibody (LTF-2, BioXcell) were injected intraperitoneally (250 μg per mouse) on day 13 (16 h before infection) and on day 14 (1 h after infection). Animal studies were approved by the Ethical Committee of Animal Experimentation (CEEA-CNB) of the Centro Nacional de Biotecnologia (CNB-CSIC, Madrid, Spain) and by Institutional Committee of the Cantonal Veterinary (Commissione cantonale per gli esperimenti sugli animali, Ticino), in accordance with national and international guidelines and the Royal Decree (RD 1201/2005) (permit n° 12013, 13013) and with the Swiss Federal Veterinary Office guidelines (permit n° TI28/17, TI02/14, and TI07/13).

### Cell and viruses

African green monkey kidney cells (BSC-40; American Type Culture Collection) and primary chicken embryo fibroblast (CEF) cells from fertilized eggs (MSD Animal Health, Salamanca, Spain) were grown in DMEM supplemented with 10% (vol/vol) FCS. All viruses were grown in primary CEF cells, purified through two 36% (w/v) sucrose cushions, and titrated by immunostaining plaque assay in BSC-40 cells as described^47^.

### Peptides

The HIV-1 peptides Pol-1 (LVGPTPVNI) and Pol-2 (YYDPSKDLI) are H-2^d^-restricted CTL epitopes^23^ and were provided by the CNB-CSIC Proteomics Service.

### Flow cytometry

Peritoneal exudate cells (PEC), peripheral blood, and spleen (SP) obtained from infected and PBS-injected mice were stained with anti-Ly6B.2 (7/4) (abcam), anti-Ly6G (1A8), -CD11b (M1/70), -CD49d (R1-2), -CD29 (HMβ1-1), -CD182 (CXCR2; SA044G4), -CD184 (CXCR4; L276F12), -CD121 (IL-1R; JAMA147), -CD45 (30-F11), -TCRβ (H57-597), -CD4 (GK1.5), -CD8 (53-6.7), -Ly6C (HK1.4), -B220 (RA3-6B2), and NK1.1 (PK136) (all from Biolegend). For intracellular cytokine staining (ICS), erythrocyte-depleted splenocytes were resuspended in RPMI 1640 with 10% FCS and 1 μg/ml Golgiplug (BD), monensin (eBioscience), and anti-CD107a (1D4B) (BD). After re-stimulation with HIV-1 peptides (6 h, 37°C, 5% CO_2_), splenocytes were stained for surface markers with anti-CD3 (145-2C11), -CD4 (GK1.5), and -CD8 (53-6.7) (all from BD), fixed, permeabilized (Cytofix/Cytoperm kit; BD), and stained intracellularly with anti-IFN-γ (XMG 1.2) and -TNF (MP6-X722) (all from BD). Cells were acquired using a GALLIOS (Beckman Coulter) or LSRII (BD) flow cytometer and data were analyzed with FlowJo software v.9.9.5 and 10.5.3.

### Immunofluorescence

Mice were anesthetized with a mixture of ketamine (100 mg/kg bodyweight, Parke Davis) and xylazine (10 mg/kg bodyweight, Bayer) and perfused with a fixative solution made of 10 mL of 0.05 M phosphate buffer containing 0.1 M L-lysine, 4% paraformaldehyde, and 2 mg/mL NaIO_4_ at pH 7.4 (PLP). After collection, spleens were incubated in 30% sucrose phosphate-buffered solution overnight. Tissues were snap frozen in O.C.T. compound (VWR Chemicals Leuven). 30 mm sections were cut with a microtome and blocked with proper sera. Sections were stained with anti-CD21/CD35 (CR2/CR1) (7E9), -IgD antibody (11-26 c.2a), -Ly-6G antibody (1A8), -CD8a (53-6.7), and -CD3ε antibody (145-2C11) (all from Biolegend). Immunofluorescence was performed using a Leica TCS SP5 confocal microscope (Leica Microsystems) using sequential acquisition to limit the signal crosstalk. Images were analyzed using Imaris software (Bitplane AG).

CD8^+^ cells were segmented with Imaris surface segmentation to determine their positions. CD8^+^ cell density was calculated with the kernel density estimate function of “scikit-learn” python package. The follicles were identified by thresholding the density function to exclude isolated CD8^+^ cells. The nearest neighbor distance of the aggregated CD8^+^ cells to the Ly6G^+^ neutrophils was obtained with a custom made R script. The *p*-value between the distributions was calculated with “ttest_ind” function of SciPy package.

### Multi-photon intravital microscopy

Intravital microscopy of GFP^+^ neutrophils and CellTrace™ Violet-labeled cognate CD8^+^ T cells in the spleen was performed in C57/BL6J mice from 4 to 5 h after i.p vaccination. 5 × 10^6^ of isolated GFP^+^ neutrophils were i.v. injected at the time of the vaccination whereas 5 × 10^6^ of labeled cognate CD8^+^ T cells were i.v injected 16 h before vaccination. Mice were anesthetized with a mixture of ketamine (100 mg/kg bodyweight, Parke Davis) and xylazine (10 mg/kg bodyweight, Bayer). Once anesthetized, mice were kept on a specific customized surgical board over a heated plate or a heated surgical bench set at 37 °C for all the time of the surgery. Hair from the left dorsal side of the animal was removed using an electric razor and a depilatory cream. Spleen was exposed through a small incision in the skin and musculature at the left dorsal side of the animal. Once out, two drops of special surgical glue were placed in the extremes of the longitudinal axis of the spleen to fix the organ on the surface of the coverslip. Finally, the organ was covered with a few drops of pre-heated PBS and petroleum jelly to guarantee proper hydration to the organ.

Deep tissue imaging was performed on a customized inverted two-photon platform (TrimScope, LaVision BioTec). Two-photon probe excitation and tissue second-harmonic generation (SHG) were obtained with a set of two tunable Ti:sapphire lasers (Chamaleon Ultra I, Chamaleon Ultra II, Coherent) and an optical parametric oscillator that emits in the range of 1010–1340 nm (Chamaleon Compact OPO, Coherent), with output wavelength in the range of 690–1080 nm. During the acquisition a temperature-controlled incubation chamber, and a 25X/NA 1.1 water immersion objective was used. The photomultipliers (PMT) used for image acquisition were either hybrid detectors or high sensitivity GaAsP. For the in vivo analysis of cell movement, two-photon micrographs were acquired in full Z stacks of 40 nm every 30 s.

Cell detection, tracking, and volumetric reconstruction were performed using Imaris (Oxford Instruments, v7.7.2). Raw data were processed and analyzed with a custom Matlab script. Distinct neutrophil subsets were differentiated based on Volume Mean (Nα < 1000 μm^3^ and Nβ > 1500 μm^3^). Cell tracks were generated semi-automatically. Tracks with duration less than 300 s were excluded from the analysis.

Measurements of cell motility were computed using the Imaris software (Oxford Instruments, v9.2) and a custom Matlab script: track duration (time interval between the first and the last time points in which a cell is tracked), track length (total length of the cell trajectory), track speed mean (track length/track duration), track displacement (length of the vector from the first to the latest centroid position of the cell), directionality (straightness of a cell track, ranging from 0 to 1: from poorly directional to highly directional), arrest coefficient (the percentage of time in which a cell moves below a threshold of 2 μm/min ranging from 0 to 1: from always moving to never moving), and differential distance (the distance variation of each neutrophil towards the closest CD8^+^ T cell). The association of the motility patterns of each neutrophil to the cell actions “patrolling” and “arrested” were determined as previously described^25^.

### Cell isolation

GFP^+^ neutrophils were purified from bone marrow of UBC (ubiquitin C)-GFP mice using a three-layer Percoll gradient of 78, 69, and 52% as previously described^48^. Neutrophils were collected at the 69–78% interface and with purity >90% injected. Cognate CD8^+^ T cells were obtained from spleens of WT mice that were DNA intramuscularly primed and NYVAC-C Δ3 i.p. boosted two weeks before. Cognate CD8^+^ T cells were purified by negative selection using the The EasySep™ Mouse CD8^+^ T Cell Isolation Kit (STEMCELL Technologies) and labeled with 5 μM of CellTrace™ Violet dye (Thermofisher).

### Low-input RNA-sequencing

Nα and Nβ neutrophils from peritoneal washes of infected mice were FACS sorted based on their expression of CD11b and Ly6G and FSC/SSC characteristics (Supplementary Fig 1a). Up to 1000 cells of each subtype were directly sorted in a 96-well plate containing Lysis Buffer and RNase Inhibitor. One sample was excluded for technical reason. cDNA amplification was performed directly from lysed cells, with no previous RNA isolation, using the SMART-Seq v4 Ultra Low Input RNA Kit (Takara). Amplified cDNA was used for preparing index tagged sequencing libraries using the Nextera XT kit (Illumina). Average library size and size distribution were determined using the 2100 Bioanalyzer High Sensitivity DNA chip (Agilent). Libraries were quantified with the Qubit fluorometer using the dsDNA HS assay (ThermoFisher Scientific). Libraries were normalized and sequenced as a pool in one lane of a Rapid flow cell in the HiSeq2500 sequencer (Illumina) to generate 61 bases single-end reads. FastQ files were obtained using the bcl2fastq v2.20.0.422 software (Illumina).

### RNA sequencing data analysis

Read quality was determined with the application FastQC v0.11.5. (‘https://www.bioinformatics.babraham.ac.uk/projects/fastqc/‘). For data analysis, sequencing adaptor contaminations were removed from reads using Cutadapt v1.7.1 and the resulting reads were mapped on the transcriptome (GRCm38 Ensembl gene-build 84) and quantified using RSEM v1.2.20^49^. Only genes with at least 1 count per million in at least 6 of the initial samples were considered for statistical analysis. The R package limma v3.46.0^50^ was used to normalize the estimated counts from RSEM and to test differential expression using the moderated *t* statistics^51^. Raw and Benjamini–Hochberg adjusted *p*-values (FDR) were calculated for each of the tests. An adjusted *p* value of less than 0.05 was considered statistically significant.

### Statistical analysis

A two-tailed Student’s *t*-test was used for comparisons between two groups, and a one-way ANOVA with Tukey post hoc test (single time points) was used for comparisons across multiple groups. For statistical analysis of CD8 T-cell response to HIV-1 Pol antigens, we utilized an approach that corrects measurements for medium response and calculates confidence intervals and *p* values of hypothesis tests^52^. Background levels (splenocytes in RPMI) were subtracted from all values used.

### Reporting summary

Further information on research design is available in the Nature Research Reporting Summary linked to this article.

## Supplementary information

Supplementary Information

Supplementary Video 1

Supplementary Video 2

Supplementary Video 3

Supplementary Video 4

Reporting Summary

## Data Availability

All datasets generated during the current study are available from the corresponding authors upon reasonable request. Neutrophils bulk RNAseq data are available as a super-series in GEO under the accession number GSE165227.
