# Supplementary material for: Neutrophil subtypes shape HIV-specific CD8 T-cell responses after vaccinia virus infection
Source: NPJ Vaccines. 2021 Apr 12;6:52. doi: 10.1038/s41541-021-00314-7 (PMC8041892; doi:10.1038/s41541-021-00314-7)
Supplement: Supplementary file 1 — Supplementary Information [file 41541_2021_314_MOESM1_ESM.pdf]

Supplementary Information

Neutrophil Subtypes Shape HIV-specific CD8 T Cell Responses After Vaccinia Virus Infection.

Mauro Di Pilato[1,2,3,4,5], Miguel Palomino-Segura[1,6]*, Ernesto Mejías-Pérez[2,7], Carmen E Gómez[2], Andrea Rubio-Ponce[6,8], Rocco D'Antuono,[1,9] Diego Ulisse Pizzagalli[1,10], Patricia Pérez[1,2], Raphael Kfuri-Rubens[11], Alberto Benguría[12], Ana Dopazo[12], Iván Ballesteros[6], Carlos Oscar S. Sorzano[2], Andrés Hidalgo[6], Mariano Esteban[2#] and Santiago F. Gonzalez[1#]*

[1]Institute for Research in Biomedicine, Università della Svizzera Italiana, Bellinzona, Switzerland
[2]Department of Molecular and Cellular Biology, Centro Nacional de Biotecnología-CSIC, Madrid, Spain
[3]Center for Immunology and Inflammatory Diseases, Massachusetts General Hospital, Boston, MA, USA
[4]Harvard Medical School, Boston, MA, USA
[5]Department of Immunology, The University of Texas MD Anderson Cancer Center, Houston, TX, USA
[6]Area of Cell & Developmental Biology, Centro Nacional de Investigaciones Cardiovasculares, Madrid, Spain
[7]Max von Pettenkofer-Institute, Ludwig-Maximilians-Universität München, Munich, Germany
[8]Bioinformatics Unit, Centro Nacional de Investigaciones Cardiovasculares, Madrid, Spain
[9]Crick Advanced Light Microscopy Science and Technology Platform, The Francis Crick Institute, London, United Kingdom
[10]Institute of Computational Science, Università della Svizzera Italiana, Lugano, Switzerland
[11]Center of Integrated Protein Science Munich and Division of Clinical Pharmacology, Klinikum der Universität München, Munich, Germany
[12]Genomics Unit, Centro Nacional de Investigaciones Cardiovasculares, Madrid, Spain

**Both authors contributed equally to this work.*
[#]These authors contributed equally to this work.

Supplementary Movie Legends

Supplementary Movie 1: MP-IVM of Nα neutrophils from spleen of NYVAC-C Δ3-infected mice from 4 to 5 h post infection.

Supplementary Movie 2: MP-IVM of Nβ neutrophils from spleen of NYVAC-C Δ 3-infected mice from 4 to 5 h post infection.

Supplementary Movie 3: MP-IVM of Nα neutrophils and cognate CD8 T cells from spleen of NYVAC-C Δ3-infected mice from 4 to 5 h post infection. Circle in yellow highlights neutrophil and CD8 T cell interaction.

Supplementary Movie 4: MP-IVM of Nβ neutrophils and cognate CD8 T cells from spleen of NYVAC-C Δ3-infected mice from 4 to 5 h post infection. Circles in yellows highlight neutrophil and CD8 T cell interactions.

Supplementary figures and legends

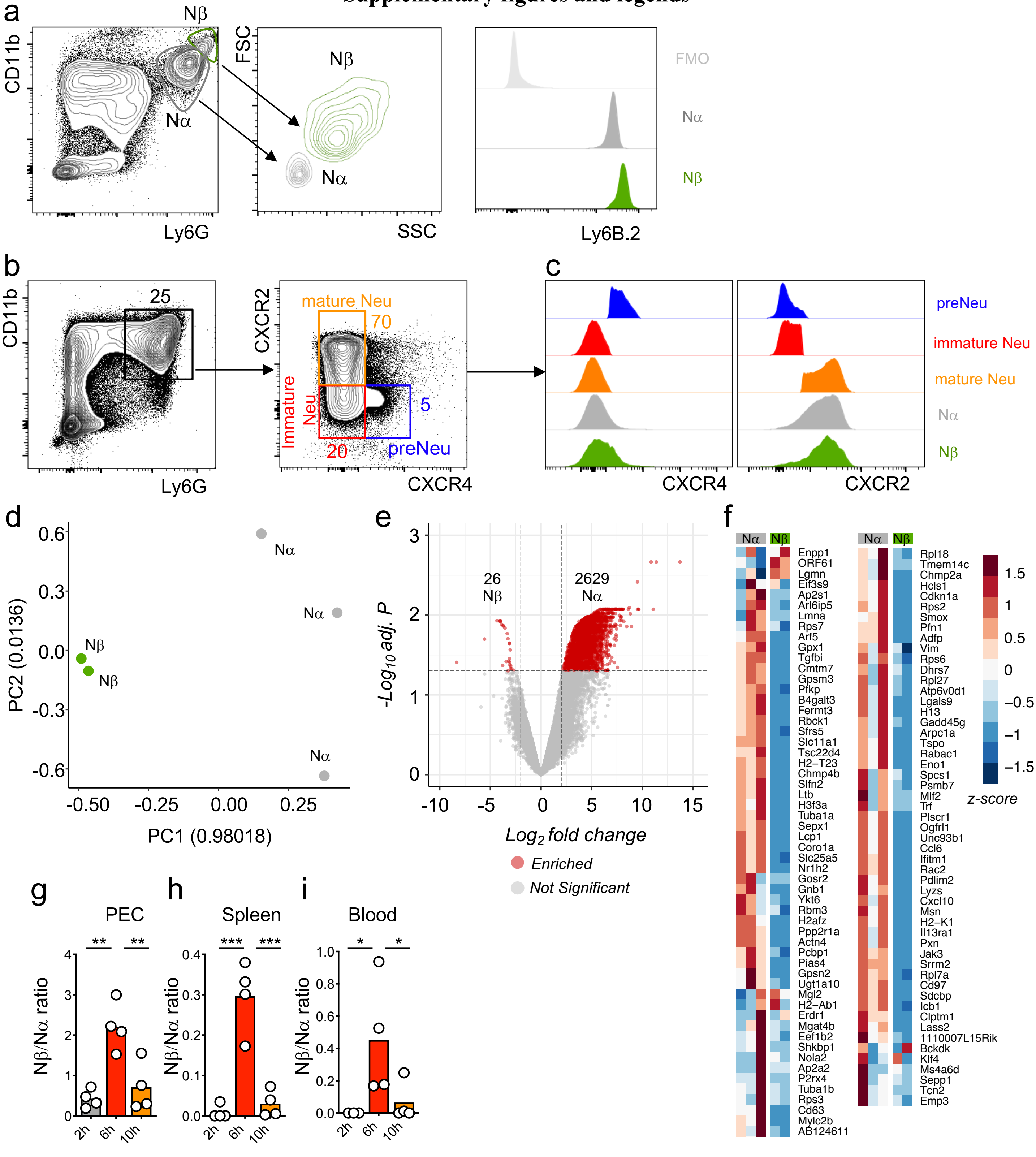

Supplementary figure 1 : Neutrophil subsets characterization.
Contour plots of Ly6G and CD11b, forward scatter (FSC) and side scatter (SSC) and Ly6B.2 layouts of Nα (grey) and Nβ (green) neutrophil subsets at 6 h post infection from PEC of NYVAC-C Δ3-injected mice (**a**). Contour plots of Ly6G and CD11b of bone marrow cells, CXCR2 and CXCR4 of bone marrow precursor neutrophils (preNeu), immature neutrophils (immature Neu) and mature neutrophils (mature Neu) (**b**). Layouts of CXCR4 and CXCR2 of preNeu, immature Neu, mature Neu, Nα and Nβ (**c**). Principal component (PC) analysis of transcriptomes (**d**). Volcano plots depicting the number of differentially expressed genes together with $\log_2$ fold change between Nβ and Nα versus $\log_{10}$ adjusted *P*. (**e**). Nα and Nβ heat map of N1 and N2 expressed signature genes (**f**). Nβ/Nα ratio from PEC (**g**), spleen (**h**) and blood (**i**) of NYVAC-C Δ3-injected mice at 2, 6 and 10 h post infection. $^*P < 0.05$, $^{**}P < 0.01$, $^{***}P < 0.001$.

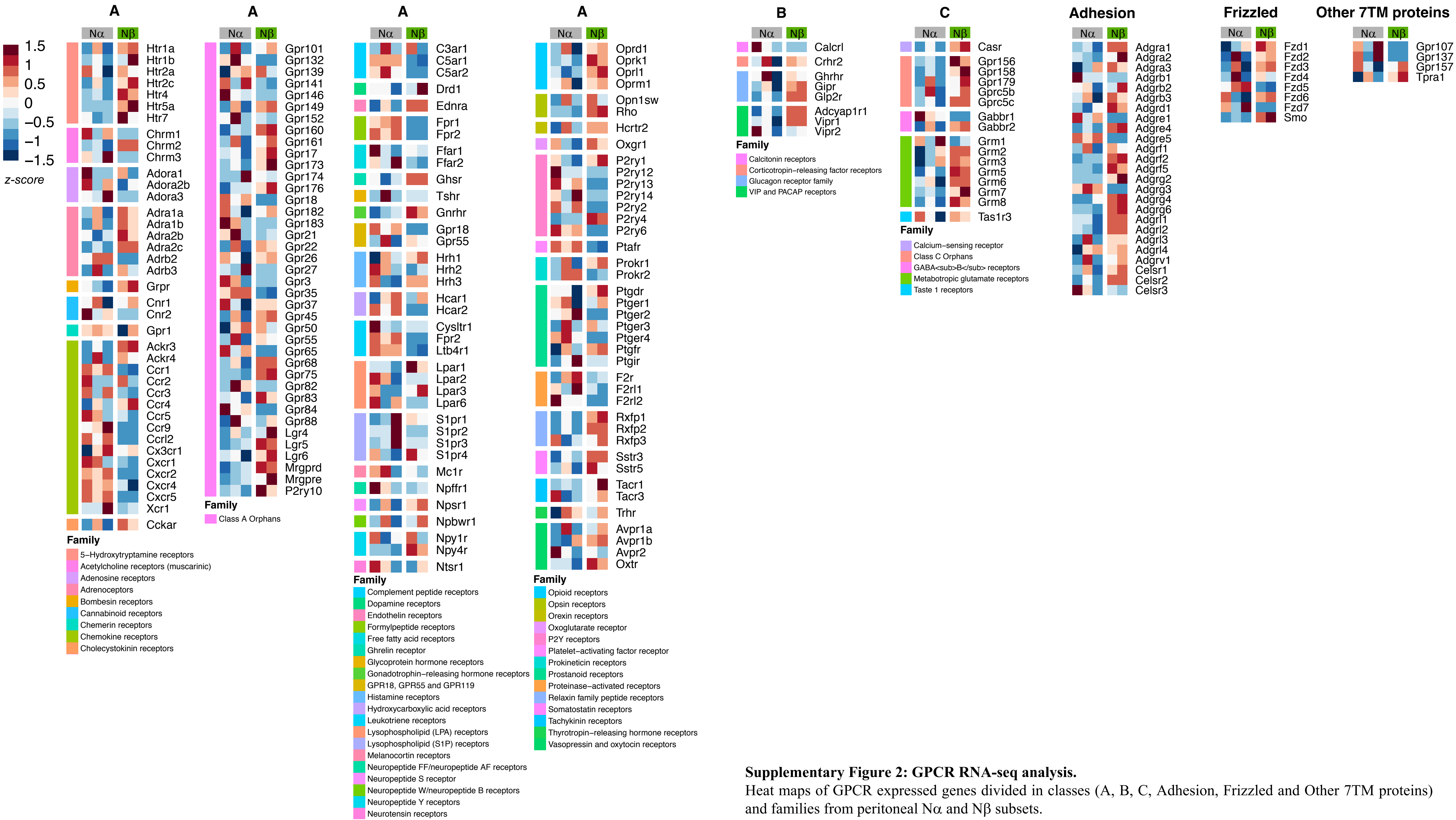

Supplementary Figure 2: GPCR RNA-seq analysis.
Heat maps of GPCR expressed genes divided in classes (A, B, C, Adhesion, Frizzled and Other 7TM proteins) and families from peritoneal Nα and Nβ subsets.
