# Supplementary material for: Neutrophil subtypes shape HIV-specific CD8 T-cell responses after vaccinia virus infection
Source: NPJ Vaccines. 2021 Apr 12;6:52. doi: 10.1038/s41541-021-00314-7 (PMC8041892; doi:10.1038/s41541-021-00314-7)
Supplement: Supplementary file 6 — Reporting Summary [file 41541_2021_314_MOESM6_ESM.pdf]

natureresearch

Corresponding author(s): Mauro Di Pilato
Santiago F. Gonzalez
Mariano Esteban
DBPR 00782

Reporting Summary

Nature Research wishes to improve the reproducibility of the work that we publish. This form provides structure for consistency and transparency in reporting. For further information on Nature Research policies, see Authors & Referees and the Editorial Policy Checklist.

Statistical parameters

When statistical analyses are reported, confirm that the following items are present in the relevant location (e.g. figure legend, table legend, main text, or Methods section).

n/a	Confirmed	
☐	☒	The exact sample size (*n*) for each experimental group/condition, given as a discrete number and unit of measurement
☐	☒	An indication of whether measurements were taken from distinct samples or whether the same sample was measured repeatedly
☐	☒	The statistical test(s) used AND whether they are one- or two-sided<br>*Only common tests should be described solely by name; describe more complex techniques in the Methods section.*
☒	☐	A description of all covariates tested
☐	☒	A description of any assumptions or corrections, such as tests of normality and adjustment for multiple comparisons
☐	☒	A full description of the statistics including central tendency (e.g. means) or other basic estimates (e.g. regression coefficient) AND variation (e.g. standard deviation) or associated estimates of uncertainty (e.g. confidence intervals)
☒	☐	For null hypothesis testing, the test statistic (e.g. *F*, *t*, *r*) with confidence intervals, effect sizes, degrees of freedom and *P* value noted<br>*Give P values as exact values whenever suitable.*
☒	☐	For Bayesian analysis, information on the choice of priors and Markov chain Monte Carlo settings
☒	☐	For hierarchical and complex designs, identification of the appropriate level for tests and full reporting of outcomes
☒	☐	Estimates of effect sizes (e.g. Cohen's *d*, Pearson's *r*), indicating how they were calculated
☐	☒	Clearly defined error bars<br>*State explicitly what error bars represent (e.g. SD, SE, CI)*

Our web collection on statistics for biologists may be useful.

Software and code

Policy information about availability of computer code

Data collection	FACSDiva software (BD Biosciences v.6.2)<br>bcl2fastq software (Illumina v.2.20.0.422)<br>LAS AF software (Leica Microsystems v.27.9723.3)<br>ImSpector software (Abberior Instruments v.16.3)
Data analysis	FlowJo software (v.9.9.5 and 10.5.3)<br>GraphPad Prism software (v.6,7 and 8)<br>Imaris software (Bitplane AG)(Oxford Instruments, v.9.2)<br>FastQC software (v.0.11.5)<br>Cutadapt software (v1.7.1)<br>RSEM (v1.2.20)<br>Limma package in R (v3.46.0)

For manuscripts utilizing custom algorithms or software that are central to the research but not yet described in published literature, software must be made available to editors/reviewers upon request. We strongly encourage code deposition in a community repository (e.g. GitHub). See the Nature Research guidelines for submitting code & software for further information.

Data

Policy information about availability of data

All manuscripts must include a data availability statement. This statement should provide the following information, where applicable:

- Accession codes, unique identifiers, or web links for publicly available datasets
- A list of figures that have associated raw data
- A description of any restrictions on data availability

All datasets generated during the current study are available from the corresponding authors upon reasonable request. Neutrophils bulk RNAseq data are available as a super-series in GEO under the accession number GSE165227.

Field-specific reporting

Please select the best fit for your research. If you are not sure, read the appropriate sections before making your selection.

☒ Life sciences ☐ Behavioural & social sciences ☐ Ecological, evolutionary & environmental sciences

For a reference copy of the document with all sections, see nature.com/authors/policies/ReportingSummary-flat.pdf

Life sciences study design

All studies must disclose on these points even when the disclosure is negative.

Sample size	No statistical methods were used to predetermine sample size, which were chosen based on prior experience with the same experimental design. Sample size was reported in the figure legends.
Data exclusions	1 sample was excluded from RNA sequencing analysis due to contamination of the sorted cell population.
Replication	All experiments were repeated at least 2 or 3 times with similar results.
Randomization	No randomization was used in this study. Mice were distributed in similar size groups under the same conditions at the start of each experiments.
Blinding	No blinding was performed for animal studies.

Reporting for specific materials, systems and methods

Materials & experimental systems

n/a	Involved in the study	
☒	☐	Unique biological materials
☐	☒	Antibodies
☒	☐	Eukaryotic cell lines
☒	☐	Palaeontology
☐	☒	Animals and other organisms
☒	☐	Human research participants

Methods

n/a	Involved in the study	
☒	☐	ChIP-seq
☐	☒	Flow cytometry
☒	☐	MRI-based neuroimaging

Antibodies

Antibodies used

Anti-Mouse CD11b-PE/Cy7 (M1/70), 101216, BioLegend;
Anti-Mouse CD11b-FITC, (M1/70), 101215, BioLegend;
Anti-Mouse CD4-APC/Cy7 (GK1.5), 100414, BioLegend;
Anti-Mouse CD4-APC/Cy7 (GK1.5), 561830, BD;
Anti-Mouse CD3- PE/Cy7 (145-2C11),561100, BD;
Anti-Mouse CD45-AlexaFluor700 (30-F11), 103128, BioLegend;
Anti-Mouse CD45- PerCP/Cy5.5 (30-F11), 103132, BioLegend;
Anti-Mouse CD8a-BrilliantViolet510 (53-6.7), 563068, BD;
Anti-Mouse CD8a-PerCP/Cy5.5 (53-6.7), 100733, BioLegend
Anti-Mouse CD90.2-AlexaFluor700 (30-H12), 105320, BioLegend;

	Anti-Mouse CD90.2-BrilliantViolet421 (30-H12), 105341, BioLegend;<br>Anti-Mouse CD90.2-PE/Cy7 (30-H12), 105325, BioLegend;<br>Anti-Mouse CD90.2-APC (30-H12), 105311, BioLegend;<br>Anti-Mouse CD90.2-Pacific Blue (53-2.1), 104306, BioLegend;<br>Anti-Mouse F4/80-APC (BM8), 123116, BioLegend;<br>Anti-Mouse F4/80-BrilliantViolet510 (BM8), 123135, Biolegned;<br>Anti-Mouse H-2Kb-PerCP/Cy5.5 (AF6-88.5), 116516, BioLegend;<br>Anti-Mouse IFN-gamma-PE (XMG1.2), 505808, BioLegend;<br>Anti-Mouse IFN-gamma-PE (XMG1.2), 554412, BD;<br>Anti-Mouse IFN-gamma-PE/Cy7 (XMG1.2), 505826, BioLegend;<br>Anti-Mouse Ly-6C-PerCP/Cy5.5 (HK1.4), 128012, BioLegend;<br>Anti-Mouse Ly-6C-APC/Cy7 (HK1.4), 128025, Biolegend<br>Anti-Mouse TNF-AlexaFluor647 (MP6-XT22), 506314, BioLegend;<br>Anti-Mouse TNF-AlexaFluor647 (MP6-XT22), 557730, BD;<br>Anti-Mouse TNF-BrilliantViolet605 (MP6-XT22), 506329, BioLegend;<br>Anti-Mouse TruStain fcX (93), 101320, BioLegend;<br>Anti-mouse Ly-6G-APC (1A8), 127613, Biolegend;<br>Anti-mouse/human CD45R/B220-APC (RA3-6B2), 103212, BioLegend;<br>Anti-mouse CD11c-FITC (N418), 117305, BioLegend;<br>Anti-mouse NK-1.1-PE/Cy7 (PK136), 108713, BioLegend;<br>Anti-mouse Ly6B.2-FITC (7/4), 53453, Abcam;<br>Anti-mouse CD49d-AlexaFluor647 (R1-2), 103613, BioLegend;<br>Anti-mouse CD29-PerCP/Cyanine5.5 (HMb1-1), 10227, BioLegend;<br>Anti-mouse CD182 (CXCR2)-PE (SA044G4), 149303, BioLegend;<br>Anti-mouse CD184 (CXCR4)-BrilliantViolet421 (L276F12), 146511, BioLegend;<br>Anti-mouse CD121 (IL-1 R, Type I/p80)-PE (JAMA147), 113505, BioLegend;<br>Anti-mouse TCRb-BrilliantViolet510 (H57-597), 109233, BioLegend;<br>Anti-Mouse CD107a-FITC (1D4B) 553793, BD.
Validation	All antibodies are commercially available and validated by previous studies done by others or our laboratory.

Animals and other organisms

Policy information about studies involving animals; ARRIVE guidelines recommended for reporting animal research

Laboratory animals	C57BL/6 or BALB/c mice were age (6-10 weeks)- and gender-matched, bred in house or acquired from Harland and Janvier labs.
Wild animals	This study did not involve wild animals.
Field-collected samples	No samples collected from the field.

Flow Cytometry

Plots

Confirm that:

- [ ] The axis labels state the marker and fluorochrome used (e.g. CD4-FITC).
- [ ] The axis scales are clearly visible. Include numbers along axes only for bottom left plot of group (a 'group' is an analysis of identical markers).
- [x] All plots are contour plots with outliers or pseudocolor plots.
- [x] A numerical value for number of cells or percentage (with statistics) is provided.

Methodology

Sample preparation	Heparinized peripheral blood collected through sub-mandibular vein puncture was treated with ACK red blood cell lysis buffer.<br>LNs and spleens were passed through 40 um cell strainers, followed by red blood cell lysis (spleens only).<br>Cell surface proteins were stained for 20 minutes at 4C.<br>Preceding antibody staining, dead cells where stained using the fixable viability violet dye Zombie Red (Biolegend) for 15 minutes at room temperature, followed by blocking of Fc receptors with TruStain fcX (Biolegend) for 20 minutes at 4oC.
Instrument	Cells were analyzed on LSR II, LSRFortessa or LSRFortessa X-20 flow cytometers (BD Biosciences).
Software	BD FACSDiva was used to acquire the data. Data were analyzed with FlowJo software version 9.9.5 and 10.5.3.
Cell population abundance	GFP+ neutrophils were purified a three-layer Percoll gradient of 78%, 69% and 52%. Neutrophils were collected at the 69–78% interface and with purity >90%.

Gating strategy

Each figure has an example of gating strategy. Singlets, viable cells and CD45+ cells were always identified for the analysis.

☒ Tick this box to confirm that a figure exemplifying the gating strategy is provided in the Supplementary Information.
